# Studies of Acceleration of the Human Body during Overturning and Falling from a Height Protected by a Self-Locking Device

**DOI:** 10.3390/ijerph191912077

**Published:** 2022-09-24

**Authors:** Marcin Jachowicz, Grzegorz Owczarek

**Affiliations:** Department of Personal Protective Equipment, Central Institute for Labour Protection—National Research Institute, 48 Wierzbowa Street, 90-133 Lodz, Poland

**Keywords:** fall protection equipment, falling from a height, anthropomorphic manikin, safety at work

## Abstract

The use of individual fall protection equipment is one of the most commonly applied methods of protecting workers whose worksites are located above the floor level. The safety of the user in such a situation depends on both the proper selection and correct use of such equipment. Additionally, aspects such as minimizing the free-fall distance before the fall arrest, as well as quick notification of an accident and efficient rescue operation, are important factors influencing safety. This paper presents a new testing method for fall arrest equipment using a test stand consisting of the Hybrid III 50th Pedestrian ATD anthropomorphic manikin and measuring set with three-axis acceleration transducers. The proposed method and test stand were developed for the design and testing of new fall protection devices equipped with electronic detection and alarm systems, for which it is necessary to determine acceleration limits in order to determine the alarm threshold. The proposed method is based on the measurement of accelerations that occur during tipping and falling from the height of an anthropomorphic manikin secured by a self-locking device. Two places of attachment of the measuring set with a three-axis acceleration sensor were analyzed at the waist belt of the manikin (abdomen and back). Moreover, the self-locking device lanyard was attached to the two points of the safety harnesses (the front and back point). The aim of the research was to check whether the acceleration values depend on the places of attachment of the measuring and anchored system, as well as to determine their maximum values. Acceleration values corresponding to fall arrest and tipping were analyzed. Limits of acceleration have been established in order to determine the threshold of alarm triggering. The non-parametric Mann–Whitney U test was used to check whether the location of the three-axis acceleration transducer and the position of the self-locking device lanyard attachment affect the value of the recorded acceleration. For results of acceleration measurements when testing the behavior of the manikin during fall arrest, no statistically significant differences were found. For results of acceleration measurements when testing the tipping behavior of the manikin, statistically significant differences occurred. This means that during fall arrest, the location of the three-axis acceleration transducer and the position of the self-locking device lanyard attachment do not matter. This work is a continuation of previous research on accelerations characterizing human body positions occurring during normal physical activities (ADL—activities of daily living).

## 1. Introduction

The practice of carrying out work at height in areas such as construction, energy, and mining indicates that, in many cases, it is impossible to eliminate the risk of falling from a height. Additionally, collective protection measures such as safety barriers and protective nets cannot be applied [1,2,3]. In such situations, the only method of protecting employees is the use of individual fall protection systems [4] composed of appropriately selected components. The first step towards proper protection of the employee is appropriate selection of a system that protects against falling from a height. Depending on the activity and location of the worksite, one of the following three protection systems can be used [4]:

- A system designed to arrest the fall from a height;
- A system designed for positioning when working at a height [5,6];
- A system designed to prevent the start of falling from a height [2,3].

The first of these systems is designed for workplaces where the risk of falling cannot be eliminated. Such a system has the widest application, and the correct selection of its components often poses a problem for users. In this case, the possibility of injury is the greatest, because it allows the protected person to start falling, and only then does it stop. Therefore, the energies and displacements occurring in such situations are the greatest. In addition, prolonged stay in suspension in safety harnesses after the fall arrest carries a number of health risks associated with partial impairment of circulation, among other concerns [7]. For this reason, it is very important that the evacuation should take place in the shortest possible time from the occurrence of the accident. In such cases, a device that can detect the fall arrest and alert the emergency services could be helpful. The calibration of such a device must be based, among other factors, on the introduction of acceleration threshold limits, upon the detection of which it must be activated. At the same time, the device should be insensitive to accelerations generated during normal motor activities [8,9,10,11,12,13,14,15].

The test method was used to compare values of acceleration and determine their threshold values during the fall arrest and tipping.

The results of eight test variants are presented in this paper.

### Current State of the Art

Considering fall protection in the work environment, we most often take into account a fall from a height, because it results in very serious injuries [16]. However, falls to the level at which we move are much more common (tipping over, e.g., as a result of stumbling). Analyzing the phenomenon of falls, we can see that in the first phase of loss of balance, they are identical for all types of falls. We most often encounter research works focusing mainly on the falls of the elderly to the level at which they move. It is estimated that more than one-third of adults aged 65 years and above suffer a fall that results in trauma at least once a year [17]. Among such people, approximately 55% of fall-related injuries occur when performing “everyday” activities at home, whereas another 23% occur outside, but near the house [18].

The scale of this phenomenon results in extensive research on the detection and reporting of falls among the elderly. Our research is inspired by such research results. The fact is that the mechanisms for losing balance are identical regardless of the type of fall; hence, this work will include a fall and a fall arrest.

Falls from a height on construction sites [19,20,21] and fall detection have repeatedly been the subject of scientific research and technical design [8,22,23,24,25,26]. Essentially, fall detection solutions can be divided into three main classes: wearables, devices located in the environment, and image-based devices. The first type requires that the monitored persons wear devices equipped with sensors such as accelerometers, gyroscopes, inclinometers, etc. [9,10,11,12]. The development of this type of device is possible thanks to the progressive miniaturization, reduction in accelerometer costs, and the availability of reliable wireless communication technologies [13,14,15,27].

For the purposes of safety research in many branches of industry and science, whose main task is to simulate the human body under the impact of dynamic loads, anthropomorphic manikins are used. One of the most widely used types is the Hybrid III [28]. Examples of applications of anthropomorphic manikins for research related to motor vehicles [29,30,31,32,33,34] and aviation [35] can be found in several publications.

Anthropomorphic manikins have also become very valuable tools in the study of mechanical phenomena accompanying sports [36,37,38,39]. Papers [40,41] present a methodology for testing dangerous factors acting on humans when tipping over on flat surfaces. An important element of that methodology was two ways of positioning the manikin before it began to fall: the first consisting of connecting the handle installed on the head of the manikin with a remote-controlled hitch, and the second consisting of the use of elastic straps located under the trunk and legs of the manikin.

The Hybrid II manikin was also used to test individual fall protection equipment [42]. The research carried out at the BIA Institute in Germany concerned the prevention of the fall of the manikin in an upright position in the absence of contact with elements of the worksite.

## 2. Materials and Methods

The Hybrid III 50th anthropomorphic manikin used for the research is a version of the Pedestrian ATD type manufactured by Humanetics [19]. Its structure allows it to simulate the behavior of the human body. It has movable joints with the ability to adjust their rigidity. In addition, the shape of the pelvis allows it to take both a standing and sitting position, thanks to which it can cooperate with safety harnesses that protect against falling from a height. This manikin has a mass of 78.15 kg and is 175 cm high [28].

The manikin was equipped with safety harnesses, to which the cord of the self-locking device was attached. In the area of the waist, a type 7267A 3-axis piezoresistive accelerometer from ENDEVCO was mounted in the outer casing, located on a rigid base. A Tektronix TDS 2004B oscilloscope was used to record and visualize the data. The system prepared in this way was used to collect acceleration measurement data in three directions perpendicular to one another. Two places of attachment of the measuring set were selected: the abdomen and back at the waist level. For the duration of the measurements, the plastic housing of the accelerometer was installed on the waist belt, which was fitted to the manikin.

Before starting the tests, a measuring system consisting of an amplifier and an oscilloscope was prepared, and then a 3-axis acceleration transducer for the waist belt was fitted to the manikin. Next, the manikin was “hung” using an electromagnetic fastening, and the measuring system was reset. The experiment started with a “layoff” electromagnetic catch followed by fall arrest by a fall arrest device. After the examination, the collected numerical data were sent to Origin and Excel software, and the acceleration values as a function of time were analyzed and visualized.

**Figure 1:**
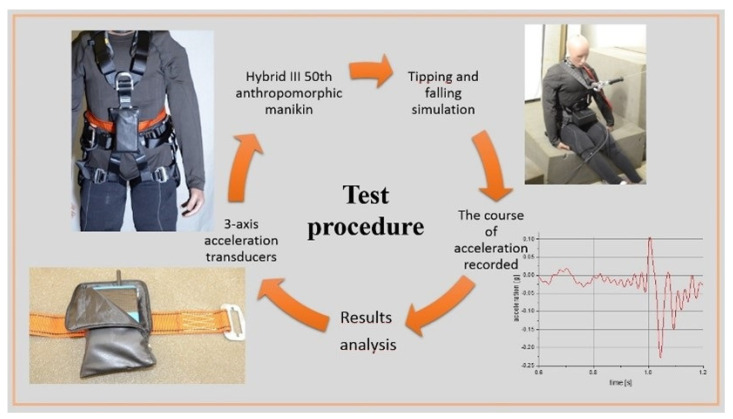
The flow of the research methodology.

Figure 1 shows the test procedure using the measurement system of the acceleration of the human body during tipping and falling from a height.

When studying the behavior of the manikin during the fall arrest, the following test variants were used:

- The self-locking device lanyard attached to the front point of the safety harnesses on the manikin, and a 3-axis acceleration transducer mounted at the abdomen of the manikin;
- The self-locking device lanyard attached to the front point of the safety harnesses on the manikin, and a 3-axis acceleration transducer mounted at the back at the level of the waist of the manikin;
- The self-locking device lanyard attached to the back point of the safety harnesses on the manikin, and the 3-axis acceleration transducer mounted at the back at the level of the waist of the manikin;
- The self-locking device lanyard attached to the back point of the safety harnesses on the manikin, and the 3-axis acceleration transducer mounted at the abdomen of the manikin.

When studying the behavior of the manikin during tipping, the following test variants were used:

- The manikin positioned facing the self-locking device, the self-locking device mounted at floor level, and the 3-axis acceleration transducer mounted at the abdomen;
- The manikin positioned facing the self-locking device, the self-locking device mounted at a 1.5 m height from the floor, and the 3-axis acceleration transducer mounted at the abdomen;
- The manikin positioned with its back to the self-locking device, the self-locking device mounted at floor level, and the 3-axis acceleration transducer mounted at the back at the level of the waist of the manikin;
- The manikin positioned with its back to the self-locking device, the self-locking device mounted at a 1.5 m height from the floor, and the 3-axis acceleration transducer mounted at the back at the level of the waist of the manikin.

The designed and constructed measuring system was checked and calibrated before use [1]. The gain of the signal from the accelerometer is set in such a way that the value of 1 g corresponds to the voltage value of 50 mV.

**Figure 2:**
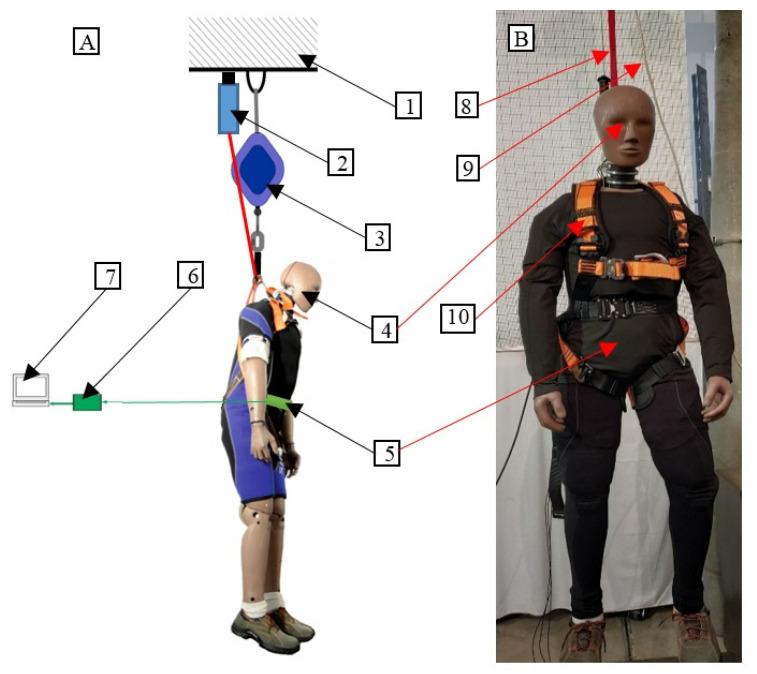
(**A**) The stand for testing the behavior of the manikin during fall arrest. (**B**) The anthropomorphic manikin prepared for testing its behavior during fall arrest: 1—rigid structure, 2—electromagnetic hitch, 3—self-locking device, 4—anthropomorphic manikin, 5—3-axis acceleration transducer, 6—data acquisition system with an analog filter and amplifier, 7—oscilloscope, 8—self-locking device lanyard, 9—safety lanyard, 10—safety harness.

The main elements of the test stand included: a horizontal steel beam in the shape of an I-beam, constituting a supporting structure; a Hybrid III anthropomorphic manikin; 3-axis acceleration transducers placed in external housing on the manikin; and a data acquisition system. The internal acceleration transducers of the anthropomorphic manikin were not used. This approach results from the fact that measuring elements mounted on the surface of the human body were used for the construction of a model of a fall arrest device at a later stage of the research. The stand prepared for studying human behavior during the fall arrest is presented in Figure 2A, and the manikin prepared for dumping is shown in Figure 2B.

**Figure 3:**
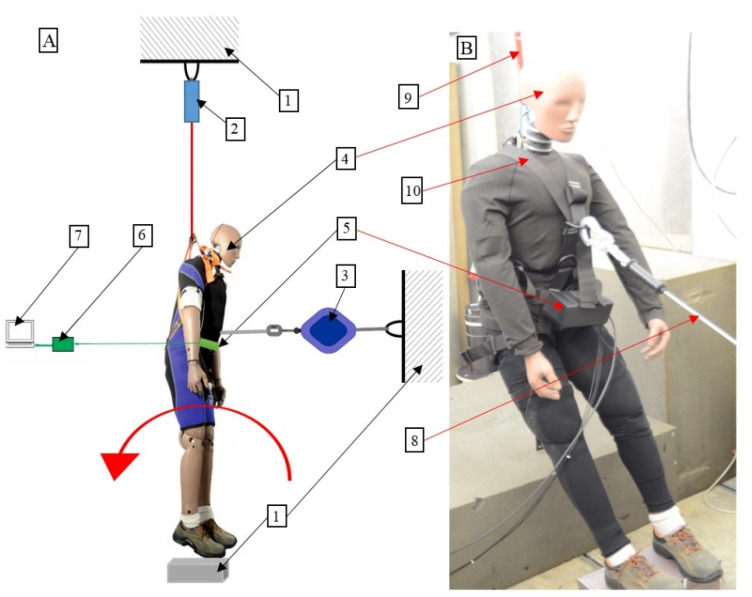
(**A**) The stand for testing the behavior of the manikin during tipping. (**B**) The anthropomorphic manikin prepared for testing tipping behavior: 1—rigid structure, 2—electromagnetic hitch, 3—self-locking device, 4—anthropomorphic manikin, 5—3-axis acceleration transducer, 6—data acquisition system with an analog filter and amplifier, 7—oscilloscope, 8—self-locking device lanyard, 9—safety lanyard, 10—safety harness. Test option: manikin facing the self-locking device attached 1.5 m above the floor level.

The stand prepared for studying the behavior of the manikin during tipping is presented in Figure 3A and the manikin prepared for tipping over is shown in Figure 3B.

## 3. Results

**Figure 4:**
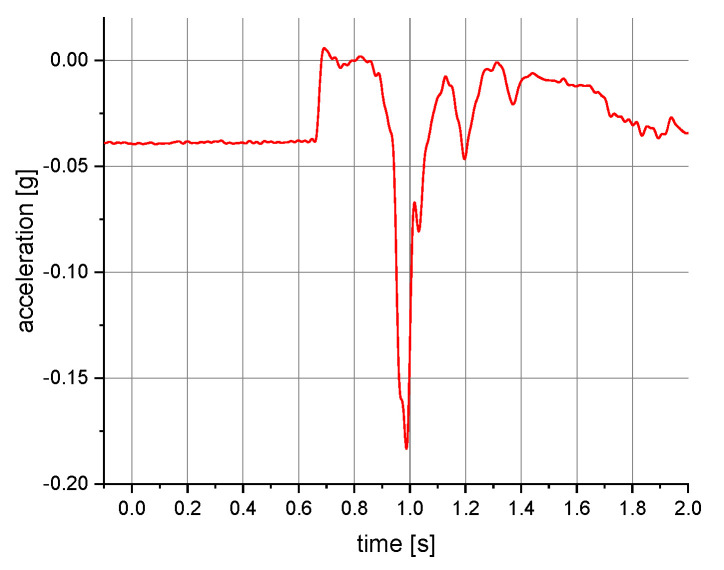
Acceleration as a function of time recorded during fall arrest. Test conditions: front point of the safety harness; the sensor mounted on the back of the manikin.

**Figure 5:**
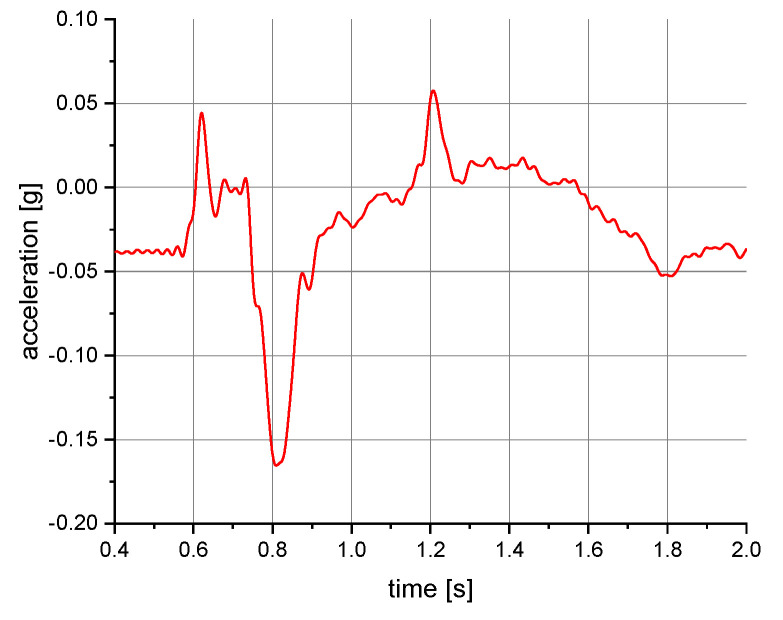
Acceleration as a function of time recorded during fall arrest. Test conditions: back point of the safety harness; the sensor mounted on the abdomen of the manikin.

**Figure 6:**
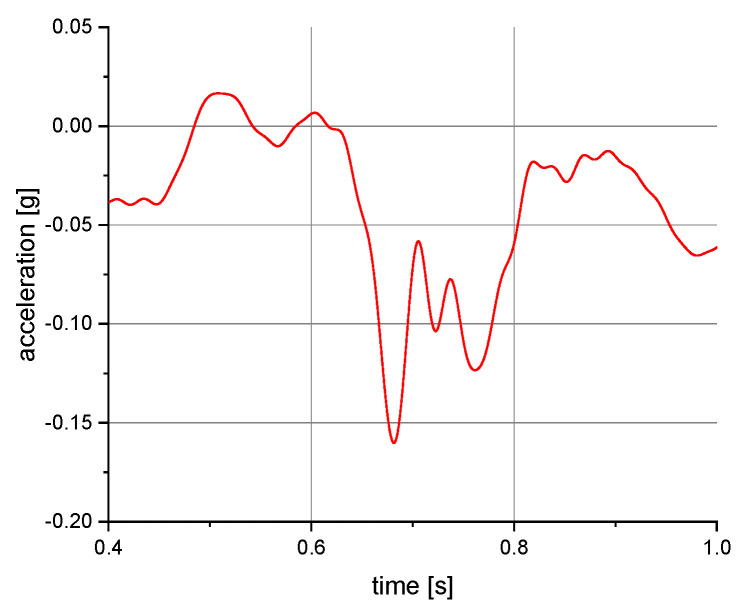
Acceleration as a function of time recorded during fall arrest. Test conditions: back point of the safety harness; the sensor mounted on the back of the manikin.

**Figure 7:**
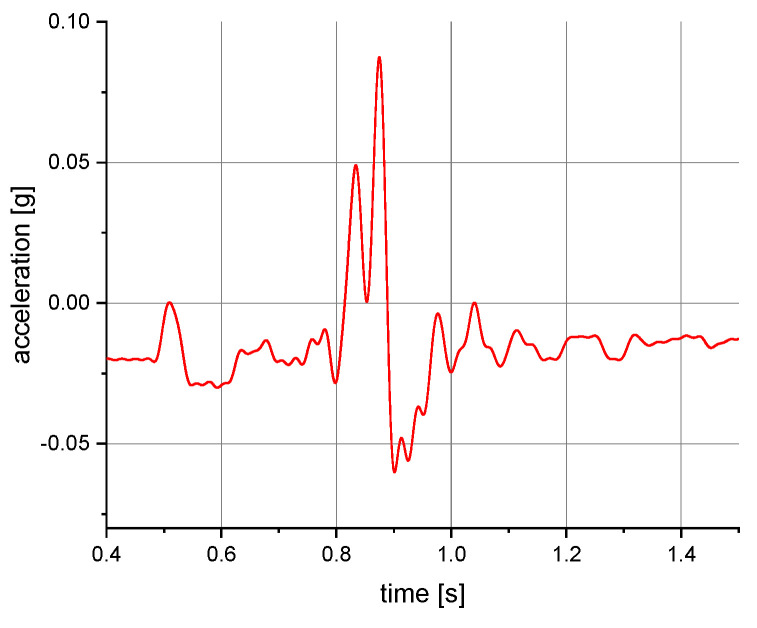
Acceleration as a function of time recorded during tipping. Test conditions: self-locking device at a height of 1.5 m above floor level; back point of the safety harness; the sensor mounted on the back of the manikin.

**Figure 8:**
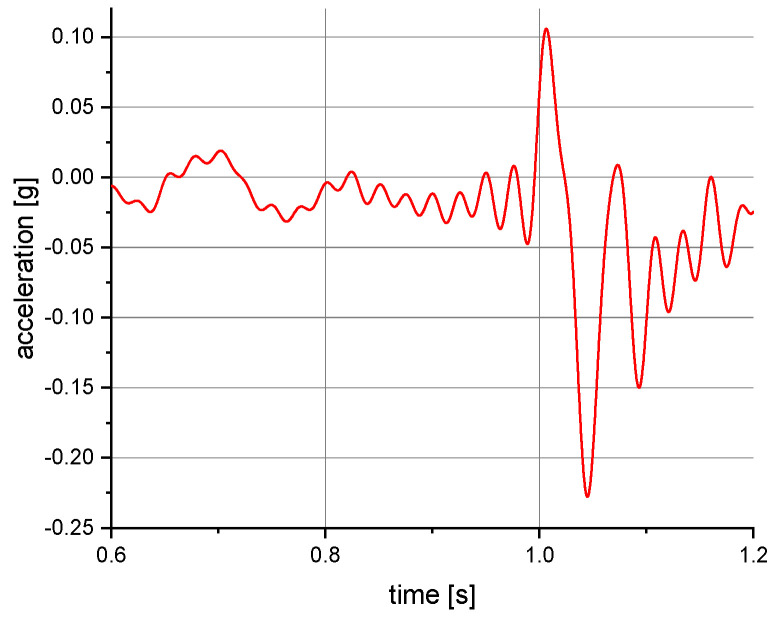
Acceleration as a function of time recorded during tipping. Test conditions: self-locking device at a level of 1.5 m above floor level; front point of the safety harness; the sensor mounted on the abdomen of the manikin.

**Figure 9:**
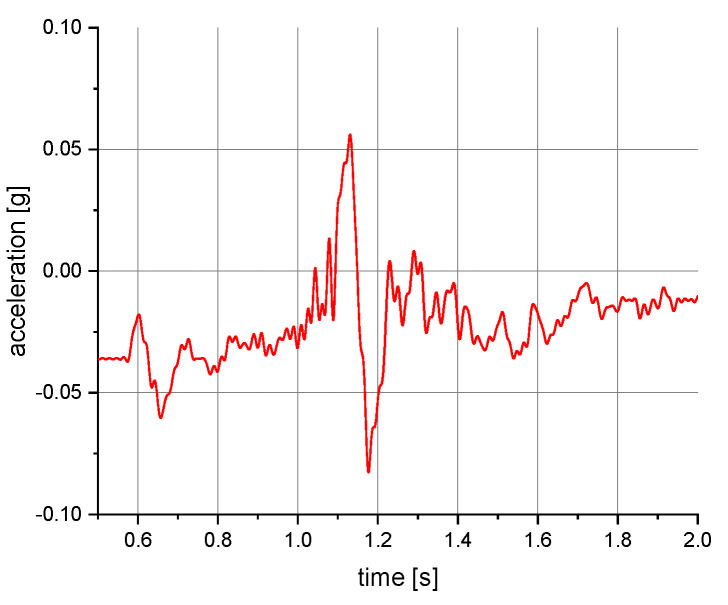
Acceleration as a function of time recorded during tipping. Test conditions: self-locking device at floor level; back point of the safety harness; the sensor mounted on the back of the manikin.

**Figure 10:**
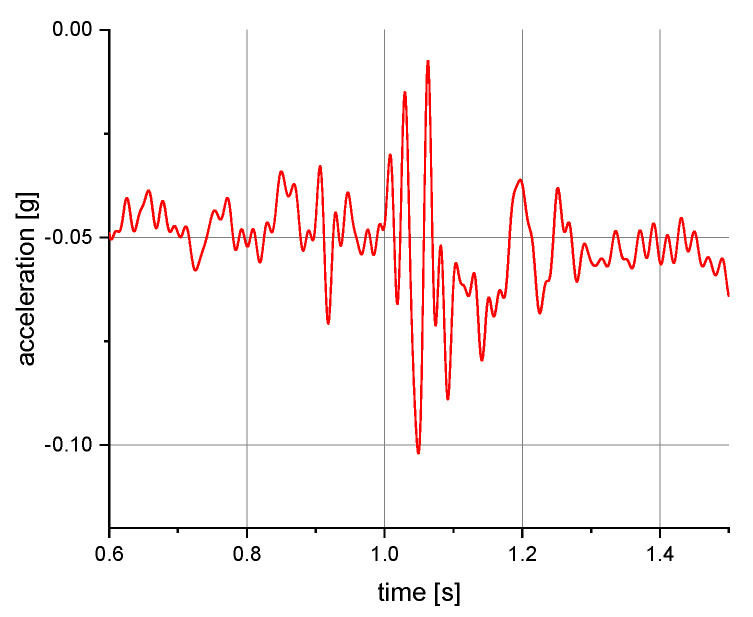
Acceleration as a function of time recorded during tipping. Test conditions: self-locking device at floor level; front point of the safety harness; the sensor mounted on the abdomen of the manikin.

The measurements of the manikin acceleration for eight variants of tests were carried out during the implementation of the above procedures. They were aimed at obtaining the values characterizing the fall arrest and tipping of the manikin. They also allowed their comparison depending on the place of attachment of the acceleration transducers and the method of testing. It was checked whether the acceleration values depend on places of attachment of the measuring and anchored system. The results take into account the value of acceleration in the vertical direction. For the other axes, the acceleration values were significantly lower than 0.01 g. A 40 or 60 Hz low-pass filter was used in the graphs below to reduce the noise. Vertical axes show acceleration values in the g unit and horizontal axes show the time of the test in seconds. The time range on the charts was limited to the registered dynamic phenomena. Examples of acceleration measurements in the vertical direction are presented in Figure 4, Figure 5, Figure 6, Figure 7, Figure 8, Figure 9 and Figure 10.

The results of the acceleration measurements when testing the behavior of the manikin during fall arrest and tipping for all variants of the initial conditions are presented in Table 1 and Table 2. The values were determined by 10 individual measurements for each variant.

Table 1 shows the results of measurements of acceleration while stopping the fall. The self-locking device cable was attached to the front or back attachment point of the safety harness, and the three-axis acceleration sensor on the waist belt on the abdominal or back side of the manikin. The mean values of the obtained results with the standard deviation were determined, and the maximum values of recorded accelerations were noted.

Table 2 shows the results of the measurements of the acceleration while tipping. The self-locking devices are anchored at floor level or at a height of 1.5 m above it. The rope of the device was attached to the front or back attachment point of the safety harness and a three-axis acceleration sensor on the waist belt from the side, the abdomen, or the back of the manikin. The mean values of the obtained results with the standard deviation were determined, and the maximum values of recorded accelerations were noted.

The obtained raw results from individual tests, the mean values of which are presented in Table 1 and Table 2, did not show a normal distribution. Therefore, the non-parametric Mann–Whitney test was used for their analysis. For results of acceleration measurements when testing the behavior of the manikin during fall arrest, with a level of significance of 0.05, no statistically significant differences were found. For results of acceleration measurements when testing the tipping behavior of the manikin, with a level of significance of 0.05, statistically significant differences occurred.

## 4. Discussion

The mean values of the acceleration of the manikin during fall arrest presented in Table 1 were in the range 0.177–0.181 g. The maximum value was 0.185 g. Moreover, no statistically significant differences were found with a level of significance of 0.05. This means that the method of anchoring the safety harness to the fall arrest device and the place at which the three-axis acceleration sensor is mounted have no significant effect on the obtained values.

The mean values of the acceleration of the manikin’s roll-over behavior for the floor level presented in Table 2 were within the range of 0.055–0.123 g, while for a height of 1.5 m above floor level, they were in the range of 0.075–0.105 g. In addition, the differences were significant with a level of significance of 0.05. The results also showed large differences in acceleration values depending on the place where the cable of the fall arrest device/three-axis acceleration transducer was attached. The differences were over 130% for the floor-level test conditions.

For individual test variants, the determined standard deviation indicated high repeatability and convergence of the results. The maximum values of accelerations did not exceed 0.2 g, and the standard deviation was below 0.027.

## 5. Conclusions

The presented method using the measurement system of acceleration of the human body during tipping and falling from a height allows for the determination of these values during different starting conditions. This method can be used to verify the correctness of electronic-based security systems measuring the acceleration of the human body. These systems aim to provide alerts about a potentially dangerous situation.

Moreover, the obtained results—together with the acceleration values determined during previous studies—allow for threshold parameters to determine the occurrence of a fall or stopping a person from falling from a height. The developed test stand can be used to determine boundary parameters for newly designed systems to shorten the fall path or provide information about how to stop the fall, and can be integrated with fall protection equipment.

Based on previous experience, it can be concluded that the limitations and disadvantages associated with this device should be considered. The self-locking device works well at workstations where the employee moves vertically. It is then characterized by a quick reaction time and a small impact on the human body (low forces and accelerations when stopping the fall). However, it is of limited use when its line or strap has to run through an edge of, e.g., a roof. Particular attention should also be paid when the device is anchored to flexible elements, e.g., a horizontal anchor rope. A “jumping” phenomenon may occur, which prevents you from falling from a height. This effect can be eliminated by introducing additional electronic control, which will be possible to implement using the research methods developed in this article.

## Figures and Tables

**Table 1:** Results of acceleration measurements when testing the behavior of the manikin during fall arrest.

Self-Locking Device Lanyard Attachment	Mounting Location of the 3-Axis Acceleration Transducer	Mean Value [g]	Standard Deviation [g]	Maximum Value [g]
Back point	Back	0.177	0.0042	0.185
	Abdomen	0.177	0.0041	0.185
Front point	Back	0.179	0.0039	0.185
	Abdomen	0.181	0.0039	0.185

**Table 2:** Results of acceleration measurements when testing the tipping behavior of the manikin.

Self-Locking Device Mounting Site	Self-Locking Device Lanyard Attachment	Mounting Location of the 3-Axis Acceleration Transducer	Mean Value [g]	Standard Deviation [g]	Maximum Value [g]
Floor level	Front point	Abdomen	0.123	0.0327	0.17
	Back point	Back	0.055	0.0097	0.07
1.5 m above floor level	Front point	Abdomen	0.105	0.0251	0.14
	Back point	Back	0.075	0.0207	0.10

## Data Availability

The data presented in this study are available on request from the corresponding author.
